# A Case Report of Progressive Multifocal Leukoencephalopathy (PML) in an Immunocompetent Patient

**DOI:** 10.7759/cureus.91491

**Published:** 2025-09-02

**Authors:** Kazi Subrina Nazneen, Nicole Mascarenhas, Eiman Elsheikh, SM Rezwanuzzaman, Sunil Kumar

**Affiliations:** 1 Acute Medicine, General Internal Medicine, Medway NHS Foundation Trust, Gillingham, GBR; 2 Neurology, Medway NHS Foundation Trust, Gillingham, GBR

**Keywords:** demyelinating disease, immunocompetent patient, john cunningham virus (jcv), opportunistic infection, progressive multifocal leukoencephalopathy (pml)

## Abstract

Progressive multifocal leukoencephalopathy (PML) is a rare but fatal demyelinating disorder resulting from reactivation of latent John Cunningham virus (JCV), which induces demyelinating lytic infection of oligodendrocytes. We report a case of PML in an individual without identifiable immunodeficiency who presented with insidious and progressive deficits in speech and motor function. Diagnostic workup revealed JC viral DNA in cerebrospinal fluid, and magnetic resonance imaging (MRI) demonstrated multiple asymmetric lesions within the subcortical and deep white matter. Despite administration of the immune checkpoint inhibitor pembrolizumab, the patient’s neurological status continued to decline, culminating in death. This case underscores the necessity of considering PML in the differential diagnosis of patients lacking classic risk factors when clinical presentation and neuroimaging findings are indicative of demyelinating pathology.

## Introduction

Progressive multifocal leukoencephalopathy (PML) is an uncommon but often fatal opportunistic infection of the central nervous system caused by the JC polyomavirus (JCV) [1]. JCV is a small, non-enveloped, ubiquitous double-stranded DNA virus in the Polyomaviridae family that is highly prevalent in the general population [2].

Primary infection with JCV usually occurs in childhood and is asymptomatic; indeed, up to 80-90% of adults have serological evidence of prior exposure [3]. The virus persists lifelong in a latent state (e.g., in kidneys and lymphoid tissue) and typically causes no harm in immunocompetent hosts [3]. However, if cellular immunity becomes profoundly impaired, JCV can reactivate and spread to the brain, where it infects oligodendrocytes and astrocytes, causing lytic demyelination. PML was historically most recognized in patients with impaired cellular immunity; classically, those with advanced HIV and low CD4+ counts, hematologic malignancies (e.g., lymphomas, leukemia), using immunosuppressive drugs in organ transplant, and certain rheumatologic diseases, and the use of modern immunomodulatory biologics (such as natalizumab in multiple sclerosis) [1,4]. PML in an immunocompetent patient is exceedingly rare; approximately only 25-30 cases have been reported till now [5,6].

Clinically, PML presents as a subacute, relentlessly progressive neurologic decline. The specific deficits depend on lesion location and often include motor weakness, speech/language disturbance, ataxia, and visual field cuts, typically evolving over weeks to months [7]. These clinical presentations overlap with other central nervous disorder (CNS) disorders (e.g., stroke, encephalitis, tumor), which often make the diagnosis challenging.

PML usually presents multifocal, bilateral, asymmetric patchy lesions that predominantly involve the supratentorial space, with less frequent involvement of the posterior cranial fossa or brainstem [8]. On CT, lesions classically appear hypodense within the periventricular and subcortical white matter. MRI is more sensitive and can reveal features earlier in the disease course [8]. In most cases, PML lesions do not enhance and produce minimal to no mass effect; however, with enhancement, it may indicate improved immunity. Further cerebrospinal fluid (CSF) analysis and brain biopsy (if necessary) are used to establish the diagnosis [8,9].

From a management perspective, there is no established antiviral therapy; management is supportive and mostly based on the immune status of the patient. Management strategies include excluding occult immunosuppression, rapid initiation/optimization of effective combination antiretroviral therapy (in HIV associated cases) [10], and consideration of immune-enhancing strategies, e.g., programmed cell death protein 1 (PD-1) inhibitors such as pembrolizumab, where no detectable immunosuppressive condition is present [11]. Overall prognosis is very poor, and median mortality is approximately 2-6 months [12].

Here, we present a case of PML in an older adult without any typical risk factors, highlighting the diagnostic challenges and management considerations in this unusual scenario. This case report underscores that PML can arise without overt immunosuppression and should be considered as a differential diagnosis even in non-traditional hosts presenting with unexplained, subacute focal neurological features, as earlier recognition may expedite the suitable interventions.

## Case presentation

The patient was a 71-year-old Caucasian male (ex-smoker), with a history of transient ischemic attack (in 2012), left lower limb amputation (due to accidental injury) with prosthetic leg, type 2 diabetes mellitus (latest HBA1c was found 63 mmol/mol in December 2024 and average controlled blood sugar ranging from 7 to 14 mmol/L on oral hypoglycemic medication with no history of insulin use), atrial fibrillation, and iron deficiency anemia. He also underwent abdominal aortic aneurysm repair in September 2024 with an endovascular stent graft, complicated by post-operative sepsis requiring three days in intensive care, from which he made a full recovery.

He was admitted to the hospital initially in January 2025 because he developed progressive speech disturbance worsening over the past 2-3 weeks. The patient demonstrated difficulty in following complex commands, although the capability to follow simple instructions remained intact. His speech was occasionally understandable; however, it was often challenging to comprehend, with significant word-finding difficulties and an element of comprehension impairment (dysphasia). Initially, there were no features of any limb weakness and no other focal neurological deficits on clinical examinations.

**Figure 1:**
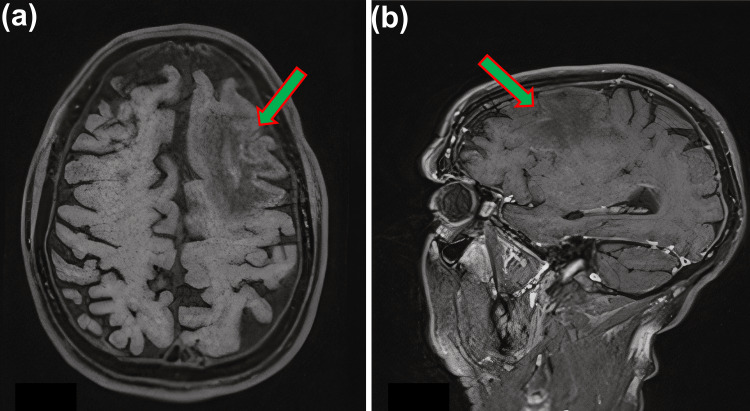
MRI images (a) T1 image without contrast, and (b) T1 image with contrast (sagittal view). These MRI images show a focal signal abnormality predominantly in the left frontal lobe white matter with subjective mild contrast enhancement within a left high frontal sulcus.

**Figure 2:**
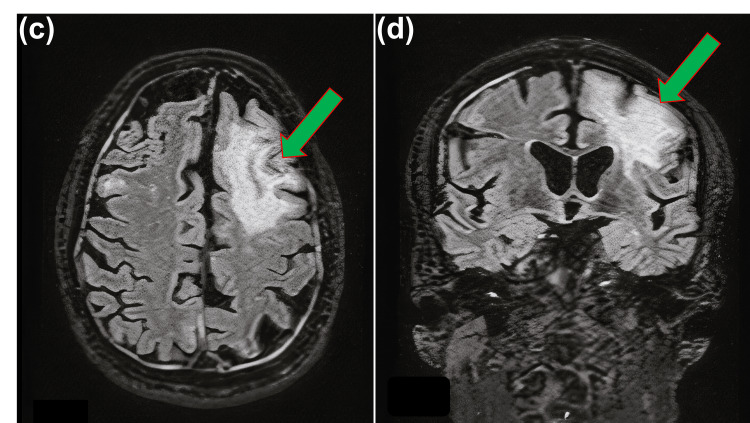
MRI images (c) axial flair, and (d) coronal flair. Axial and coronal flair showing the left frontal lobe lesion as observed in Figure 1.

On presentation to the emergency department, initial MRI brain imaging revealed a focal signal abnormality in the left frontal cortex with cortical swelling and diffusion restriction (Figures 1a-1b, and Figures 2c-2d). The appearances were not typical of an acute infarct, and meningoencephalitis was considered more likely. The patient was empirically treated with intravenous acyclovir and cefotaxime initially, considering subacute neurological presentation and radiological findings.

**Figure 3:**
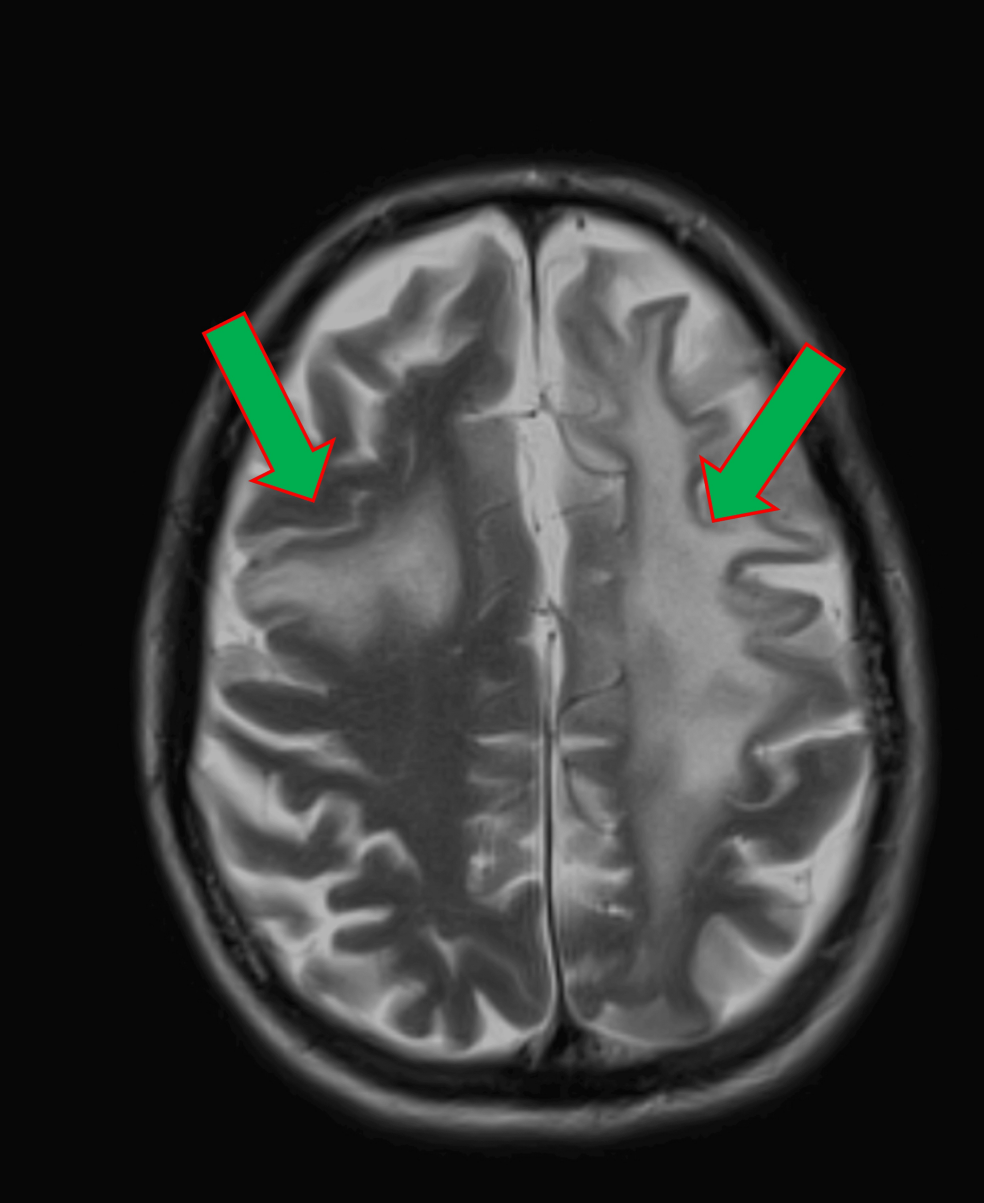
MRI T2-weighted image showing approximately a 7.4 × 3.3 cm-sized left frontal lobe white matter lesion and a smaller but similar lesion (about 1.5 cm) in the right frontal lobe.

Lumbar puncture demonstrated normal microscopy, biochemistry, and an initially negative viral polymerase chain reaction (PCR). During his ward stay, he developed gradual right-side hemiparesis (greater weakness in the right upper limb) over the next few days (around 7-10 days). He received five days of high-dose methylprednisolone as his condition was deteriorating neurologically. A repeat contrast-enhanced MRI showed about a 7.4 × 3.3 cm lesion in the left frontal white matter with subtle sulcal enhancement and a similar but smaller lesion in the right frontal lobe (Figure 3).

The case was discussed with a tertiary neurology center. In light of the progressive course and MRI appearance, PML became a diagnostic consideration - despite the patient’s immunocompetent status. A repeat CSF PCR was sent and returned positive for JC virus DNA (quantitative viral load ~132 copies/mL), confirming the diagnosis of PML (Table 1).

**Table 1:** CSF analysis findings with reference ranges for all parameters. JC: John Cunningham; NMDA: N-methyl-D-aspartate; CSF: cerebrospinal fluid

Test	Result	Units	Reference Range
Appearance	Clear & Colorless	N/A	N/A
WBC	1	Cells/cu.mm	<5
CSF glucose	5.7	mmol/L	2.2-3.9
Gram stain	Negative	N/A	N/A
Culture	No growth	N/A	N/A
Fixed cell NMDA CSF	Negative	N/A	N/A
Herpes simplex virus DNA	Not detected	N/A	N/A
Varicella zoster virus DNA	Not detected	N/A	N/A
Enterovirus RNA NOT detected	Not detected	N/A	N/A
Polyoma JC virus DNA quantification	Detected (132 JCV DNA)	Copies/mL	N/A

Meanwhile, an extensive search for an underlying cause of immune compromise was conducted. Serologies for collagen vascular diseases (antinuclear antibody (ANA), anti-neutrophil cytoplasmic antibody (ANCA)) were negative, as were anti-neuronal antibodies (paraneoplastic panel including CASPR2, N-methyl-D-aspartate receptor (NMDA-R), etc.). Serum immunoglobulin levels and complements were found within normal ranges. A CT scan of the chest, abdomen, and pelvis found no evidence of malignancy or lymphoproliferative disease. A whole-body PET scan was suggested to sensitively rule out occult cancer, but the family declined due to concerns about contrast exposure. HIV testing was reconfirmed negative, and an interferon-gamma release assay for tuberculosis was found negative. In summary, no concurrent infection, autoimmune disorder, or cancer was identified except for borderline chronic lymphopenia (Table 2). Lymphocyte subset analysis was not done as the patient was transferred to the tertiary center.

**Table 2:** Findings from laboratory blood tests with associated reference ranges. GFR: glomerular filtration rate; Hi Sens CRP: high-sensitivity C-reactive protein (HS-CRP); ANCA: anti-neutrophil cytoplasmic antibodies; IGRA: interferon-gamma release assay; IGCC: the International Federation of Clinical Chemistry

Test	Result	Units	Reference Range
WBC	12.6	10^9^/L	4.0-11.0
RBC	3.09	10^12^/L	4.50-5.50
Hemoglobin	83	g/L	130-170
Hematocrit	0.26	L/L	0.40-0.50
Platelet count	522	10^9^/L	150-410
Automated neutrophil count	11.8	10^9^/L	2.0-7.0
Automated lymphocyte count	0.4	10^9^/L	1.0-4.0
Automated mono count	0.4	10^9^/L	<1.0
Automated eosinophil count	0.0	10^9^/L	0.0-0.4
Automated basophil count	0.0	10^9^/L	0.0-0.3
Estimated GFR	>90	mL/min/1.73m^2^	
Creatinine	67	umol/L	59-104
Hi Sens CRP	9.6	mg/L	0.0-5.0
HbA1c (IFCC-aligned)	63	mmol/mol	20-41
IgG	7.6	g/L	7.0-16.0
IgA	2.10	g/L	0.70-4.00
IgM	0.81	g/L	0.40-2.30
Antinuclear antibody	Negative	N/A	N/A
ANCA pattern	Negative	N/A	N/A
CASP2	Negative	N/A	N/A
Leucine-rich glioma	Negative	N/A	N/A
Fixed cell NMDA serology	Negative	N/A	N/A
Anti-HIV 1&2 + p24Ag	Not detected	N/A	N/A
IGRA	Negative	N/A	N/A
Treponema pallidum	Not detected	N/A	N/A

Given the lack of any reversible immunosuppressive condition, the main therapeutic option discussed was immune-based therapy for PML. Based on emerging reports of success with checkpoint inhibitors in PML, the patient was transferred to a tertiary center and received pembrolizumab (an anti-PD-1 monoclonal antibody) in March 2025. He tolerated the infusion without acute complications. However, over the subsequent 2-3 weeks, there was no improvement in his neurological exam - he remained severely aphasic and later on developed bilateral weakness (the right worse than the left).

Unfortunately, during this period, he also contracted COVID-19 (mild pneumonitis) and then a hospital-acquired pneumonia. These infections, combined with his debilitated state, led to further decline. The second planned dose of pembrolizumab was put on hold as he was too medically unstable. Despite antibiotics, the patient’s condition deteriorated and sadly passed away approximately 4 months after symptom onset.

## Discussion

PML is classically an opportunistic infection seen in profoundly immunosuppressed individuals [1]. Identifying PML in an apparently immunocompetent patient is therefore challenging and often not initially high on the differential diagnosis. In reported cases of immunocompetent PML, clinicians have frequently pursued exhaustive evaluations for alternative diagnoses, including autoimmune encephalitis and malignancies, before considering PML [5,13].

In the present case, a comprehensive autoimmune panel was negative, and imaging (CT of the chest, abdomen, pelvis) revealed no evidence of malignancy, effectively ruling out autoimmune demyelination, vasculitis, and paraneoplastic processes as explanations for the patient’s brain lesions. The patient’s negative HIV test is also significant, as advanced HIV has historically been the most common predisposing condition for PML [1,13]. Likewise, a negative interferon-gamma release assay (IGRA) helped to exclude tuberculosis, which can cause immune dysfunction. The absence of any significant identifiable immunocompromising condition in this patient made the diagnosis of PML unexpected and explains why many such cases require a brain biopsy for confirmation [14]. However, MR spectroscopy can also be considered as it can provide significant information and even prevent brain biopsy [1].

In retrospect, the detection of JC virus DNA in CSF clinched the diagnosis, but only after other differentials had been excluded. This case underscores that PML, while exceedingly rare in immunocompetent hosts, must remain on the differential diagnosis when subacute progressive neurologic deficits and white matter abnormalities are present [5,13].

A key question in an ostensibly immunocompetent PML case is what underlying factors might have permitted JC virus reactivation. Careful review often reveals subtle or “occult” immune defects. In this patient, several chronic conditions and historical factors could have contributed to immune dysfunction. Chronic lymphopenia (even if not fully characterized) raises the possibility of idiopathic CD4⁺ T-lymphocytopenia - a recognized risk factor for PML in HIV-negative individuals [11]. Similarly, metabolic diseases such as poorly controlled diabetes mellitus (DM), which are associated with immune impairment, have been noted in PML patients without other immunosuppression [15]. However, the patient had moderately controlled DM on oral hypoglycemic medication with no history of insulin use and no evidence of recent macro or microvascular complications. The patient’s cardiovascular comorbidities and history of aortic aneurysm repair followed by an intensive care unit admission are also noteworthy. Major surgery and sepsis can induce transient immunosuppressive states (“immune paralysis”), potentially providing an opportunity for JC virus reactivation [16].

Notably, in one review of 38 PML cases without overt immunosuppression, 58% had no obvious underlying diagnosis - but among those, about 23% were found to have low CD4+ counts meeting criteria for idiopathic CD4⁺ T-cell lymphocytopenia [14]. These data suggest that many “immunocompetent” PML patients harbor unrecognized immune deficits. It has been postulated that some patients might experience a transient immunodeficient state (for example, due to severe illness or an unrecognized viral/immunologic syndrome) that triggers JC virus reactivation [15]. In the present case, the combination of chronic medical conditions and a history of major surgery and ICU admission could represent such permissive factors, even though the patient was not on immunosuppressive therapies. Therefore, an important lesson is to consider cumulative and subtle immunosuppressive influences (chronic diseases, age-related immunity changes, etc.) when evaluating PML in an “immunocompetent” host.

Detection of JC polyomavirus DNA in the CSF by PCR is a cornerstone of PML diagnosis. A positive CSF JCV PCR in the appropriate clinical context is considered diagnostic of PML, obviating the need for brain biopsy in most cases. Typically, JC virus (JCV) DNA quantitation cut-off values can vary significantly depending on the specific laboratory assay and the sample type. It was mentioned in a prior study that having low levels of JCV DNA (<100 copies/mL) present in CSF makes the diagnosis challenging. In the context of this, the cut-off values for PML cases can be considered as 100 copies/mL. In our case, the CSF sample was reported as positive with 132 copies/mL quantification, which is well above the considered cut-off value [17].

The test is highly specific - studies report a specificity around 95-99% [18], meaning false-positives are exceedingly uncommon. However, the sensitivity of JCV PCR is more limited, roughly 70-90% depending on the assay and cohort [19]. Early in the disease or in patients with intact immune systems, the CSF viral load may be very low. This can lead to false-negative results if the initial lumbar puncture sample is not optimally handled or if less sensitive assays are used. Ultrasensitive PCR for JC virus detection offers superior sensitivity compared to conventional PCR, especially with low viral loads when analyzing CSF. While conventional PCR may miss some infections, which could lead to false-negative results, ultrasensitive PCR is able to detect the virus even at very low concentrations. This enhanced detection capability enables earlier and more accurate diagnosis of PML [20].

In this patient, the CSF JCV PCR was found to be positive with 132 JCV DNA copies/mL, confirming the diagnosis. A negative result would have necessitated further investigation, given the high clinical suspicion. Overall, JCV PCR remains the preferred diagnostic test for PML due to its high specificity.

Neuroimaging also plays a vital role in both raising suspicion for PML and monitoring its course. Magnetic resonance imaging (MRI) is far more sensitive than CT for detecting the demyelinating lesions of PML. The radiologic hallmarks of PML are multiple T2-hyperintense, T1-hypointense demyelinating lesions in subcortical white matter, asymmetric distribution, without mass effect or significant edema [1]. When these imaging features are coupled with the clinical picture of progressive neurological deficits, they strongly point toward PML, even before virological confirmation is obtained.

In our case, serial MRI showed asymmetric, multifocal subcortical white-matter lesions - a dominant left frontal focus (7.4 × 3.3 cm) with a smaller contralateral lesion demonstrating T2/FLAIR hyperintensity with mild contrast enhancement and no substantial mass effect, evolving over days. This pattern is far more typical of PML than a vascular-territory infarct, encephalitis, or neoplasm. This case was later discussed with the tertiary center neuro radiology and was suggested for a repeat lumbar puncture for JC virus PCR for suspected PML based on clinical presentations and MRI findings, which came back as positive.

Treating PML remains challenging, as no antiviral directly targets the JC virus. When possible, reversing immunosuppression, such as starting antiretroviral therapy or withdrawing an offending drug, is the cornerstone of management. In patients without an obvious immune deficit, checkpoint inhibitor immunotherapy with pembrolizumab has been explored to restore virus-specific T-cell function. Pembrolizumab acts by blocking the programmed cell death protein 1 (PD-1) expression on lymphocytes in peripheral blood and CSF. PD-1 functions as an inhibitory immune checkpoint that may contribute to impaired antiviral responses [11].

Our patient received one dose but could not complete therapy due to an intercurrent infection, and afterwards, sadly passed away before receiving a second dose of pembrolizumab. Preliminary case series report that pembrolizumab induces clinical stabilization or improvement in roughly 60% of treated PML patients, often accompanied by reduced CSF JCV load and enhanced CD4+ and CD8+ T-cell responses [11]. However, responses are inconsistent, and immune-related complications, including immune-restriction inflammatory syndrome (IRIS), can occur. This immune-based strategy offers a compassionate use option in a disease that is otherwise often fatal, but it comes with substantial risks and remains an investigational therapy pending further evidence.

## Conclusions

PML should not be dismissed as a diagnosis in patients without classic risk factors if the clinical and radiological picture is suggestive. This case demonstrates that PML can occur in an apparently immunocompetent host, likely facilitated by subtle immunologic impairments (chronic lymphopenia, comorbid conditions, and age-related immune decline). Timely diagnosis required repeated CSF analysis and recognition of typical MRI patterns. Sadly, like many PML cases, the outcome was poor - highlighting the dire need for effective treatments. Immune-based therapies such as PD-1 inhibitors are being explored, but their efficacy is variable, and they also present some risks. Clinicians managing unexplained progressive encephalopathy should consider PML even in the absence of overt immunosuppression, as earlier expedited intervention could be possible. Continuing research into antiviral drugs, immunotherapies, and preventative strategies is essential to change the unfortunate prognosis of this disease.

## References

[1] Cortese I, Reich DS, Nath A (2021). Progressive multifocal leukoencephalopathy and the spectrum of JC virus-related disease. Nat Rev Neurol.

[2] Bellizzi A, Anzivino E, Rodio DM, Palamara AT, Nencioni L, Pietropaolo V (2013). New insights on human polyomavirus JC and pathogenesis of progressive multifocal leukoencephalopathy. Clin Dev Immunol.

[3] Wang JP, Wang ZZ, Zheng YS (2012). JC virus existence in Chinese gastrointestinal carcinomas. Oncol Lett.

[4] Glenn T, Berger JR, McEntire CR (2025). Natalizumab-associated progressive multifocal leukoencephalopathy. Front Neurol.

[5] Sriwastava S, Khan E, Khalid SH, Kaur A, Feizi P (2022). Progressive multifocal leukoencephalopathy in an immunocompetent patient: A case report and review of literature. J Med Virol.

[6] Ngo M, Tang N, Le Q (2023). Presumptive progressive multifocal encephalopathy in an immunocompetent patient: A rare case report. Cureus.

[7] (2025). Progressive multifocal leukoencephalopathy in HIV. https://emedicine.medscape.com/article/1167145-overview.

[8] Gone J, Fontaine T, Kumar G (2024). A rare case of progressive multifocal leukoencephalopathy. Radiol Case Rep.

[9] Sarbu N, Shih RY, Jones RV, Horkayne-Szakaly I, Oleaga L, Smirniotopoulos JG (2016). White matter diseases with radiologic-pathologic correlation. Radiographics.

[10] Schweitzer F, Laurent S, Cortese I (2023). Progressive multifocal leukoencephalopathy: pathogenesis, diagnostic tools, and potential biomarkers of response to therapy. Neurology.

[11] Cortese I, Muranski P, Enose-Akahata Y (2019). Pembrolizumab Treatment for Progressive Multifocal Leukoencephalopathy. N Engl J Med.

[12] Kim J, Kim C, Lee JA (2023). Long-term prognosis and overall mortality in patients with progressive multifocal leukoencephalopathy. Sci Rep.

[13] Johansen KK, Torp SH, Rydland J, Aasly JO (2013). Progressive multifocal leukoencephalopathy in an immunocompetent patient?. Case Rep Neurol.

[14] Tan CS, Koralnik IJ (2010). Progressive multifocal leukoencephalopathy and other disorders caused by JC virus: Clinical features and pathogenesis. Lancet Neurol.

[15] Meylor J, Artunduaga DC, Mendoza M, Hooshmand SI, Obeidat AZ (2024). Progressive multifocal leukoencephalopathy in patients with chronic kidney disease. Neurol Sci.

[16] Hotchkiss RS, Monneret G, Payen D (2013). Immunosuppression in sepsis: A novel understanding of the disorder and a new therapeutic approach. Lancet Infect Dis.

[17] Warnke C, von Geldern G, Markwerth P (2014). Cerebrospinal fluid JC virus antibody index for diagnosis of natalizumab-associated progressive multifocal leukoencephalopathy. Ann Neurol.

[18] Harypursat V, Zhou Y, Tang S, Chen Y (2020). JC Polyomavirus, progressive multifocal leukoencephalopathy and immune reconstitution inflammatory syndrome: a review. AIDS Res Ther.

[19] Ferretti F, Bestetti A, Yiannoutsos CT (2018). Diagnostic and prognostic value of JC virus DNA in plasma in progressive multifocal leukoencephalopathy. Clin Infect Dis.

[20] Nakano K, Kawashima A, Nakamoto T (2025). Early diagnosis of progressive multifocal leukoencephalopathy in untreated HIV infection via ultrasensitive PCR testing for JC virus: A case report. IDCases.

